# “Mentor in Bioeconomics *avant la lettre*”: A tribute to Bernard Witholt by those who worked with him

**DOI:** 10.1111/1751-7915.12293_2

**Published:** 2015-06-15

**Authors:** Auxiliadora Prieto, Gerrit Eggink, Marcel Wubbolts

**Affiliations:** 1 Polymer Biotechnology Lab, Centro de Investigaciones Biologicas (CIB)C/ Ramiro de Maeztu, 9, 28040, Madrid, Spain; 2 Wageningen University and Research Centre, AFSG6700AA, Wageningen, The Netherlands; 3 Royal DSM, CTO OfficeMauritslaan 49, 6129EL, Urmond, The Netherlands

Bernard Witholt was appointed a professor of biochemistry at the University of Groningen at the age of 30 years. He was born in 1941 in The Hague (the Netherlands), spent his early life in Brazil and moved to the USA during his teenage years. After studying biology at Amherst College (Massachusetts), he undertook a PhD at Johns Hopkins University in Baltimore. Before returning to the Netherlands, he did his post-doc and became a staff member at the Department of Biology and Chemistry at the University of California, San Diego.

In Groningen, he established a research group focusing on the interaction of proteins with membranes and their functions in microbial processes. His paper on the development of procedures for forming spheroplasts from *Escherichia coli* was much cited. During his time in the Netherlands, Bernard also worked with other researchers on the purification and analysis of cholera toxin-related heat-labile enterotoxin from *E. coli*. His scientific interest was driven by a curiosity to understand how things work, but he also felt a huge responsibility and obligation towards translating science into new opportunities for society. He was a great believer in man's resourcefulness with respect to the solving of problems and the improvement of well-being. In the early 1980s, Bernard recognized the potential and importance of modern biotechnology, and he started a new research programme working on microbial biotransformations aimed at producing chiral compounds for the chemical and pharmaceutical industries. His pioneering work in this area has been followed by many research groups and industrial laboratories, resulting in new technologies, processes and products. Bernard also developed a national programme for the Netherlands designed to stimulate biotechnological research. With visionary lectures, he convinced policymakers and companies to invest. He was a fantastic advisor for, and initiator of, numerous successful biotech start-ups, and was the founding father of the Zernike Science Park in Groningen. In 1992, he established in the Institute of Biotechnology of Swiss Federal Institute of Technology in Zürich (IBT, ETH Zurich) and worked on alkane-degrading bacteria, biocatalysis and bioplastics until 2005 when he retired.

What follows are personal tributes from a few of the many young people he mentored over his more than four decades of an exceptional research career.

Bernard was extremely successful in making the right connections with other researchers and laboratories and quickly built fruitful networks for advancing his research interests. His infectious enthusiasm was also able to attract many – very many – students to join his laboratory and undertake much research during times when funding was scarce. His workspace grew from just one laboratory to include a second, and then more; by the time I left Bernard's group in 1979, an entire wing of the building was his. Bernard was one of the first to see the great opportunities of biotechnology. He wrote position papers outlining his great vision on the subject, and eventually he made his ideas come true by shifting his research interests completely to it, securing the funds to build a group that became greatly successful in the field. Thinking of Bernard, I can see a man who was thrilled to tackle technical challenges, who loved to do calculations to test whether an assumption was real or not, who had the capacity to see everything in a big perspective, but also who had a really warm and enthusiastic personality. He gave me the opportunity to make my own choices when doing my PhD. It was great to work with him and, needless to say, he taught me to do research in a well-organized but visionary way [**Lou de Leij (1974–1979)**].

I was looking for a PhD supervisor and Bernard gladly accepted me as his pupil. It was only years later that I realized how lucky I had been to meet him at a time his agenda wasn't as busy as it was a few years later, when his students had to fight for his time and attention. Almost every day, we spent hours talking about literally everything, though mostly science, philosophy and the meaning of life. Having a conversation with Bernard meant that he was doing most of the talking. Supported by his characteristic arm gestures, he shared his views and visions – many of which I deemed overexaggerated at the time but which came true after one, two or sometimes three decades [**Harm van Heerikhuizen (1972–1977)**].

After I had finished my masters studying membranes and polyhydroxyalkanoates (PHA) in *Pseudomonas*, Bernard asked me to join his group as PhD student. The topic was genetic engineering of alkane conversion by *Pseudomonas oleovorans*, a unique opportunity, in 1982, to start working in this new area of biotechnological research. At that moment, however, I was so bold to say that I needed 2 weeks to think about his proposition. Obviously, I still had to learn a lot. My years as PhD student and post-doc in Bernard's group have been fantastic for me. In this period, I could develop my skills and expertise in an atmosphere of freedom, trust, enthusiasm and collective ambition. Discussions with Bernard were always very stimulating and he triggered me to trust my resourcefulness. I remember his visionary papers and lectures describing the use of microorganisms as biocatalysts, including the economics of the process, for the production of polymer building blocks. Today one of my PhD students is using the same strains and approaches to produce these building blocks from renewable feedstocks. The enormous positive power, generosity and scientific brilliance of Bernard have remained a source of inspiration to me all these years [**Gerrit Eggink (1980–1990)**].

The way Bernard organized and facilitated the work on hydrocarbon-utilizing microorganisms in Groningen during the 1980s was an exemplary case of ‘*Convergence avant la lettre*’. In his laboratories, people were working on genetic engineering and physiology of Pseudomonads and other, at the time, relatively inaccessible bacterial species, combined with the biochemistry of bioconversions of aliphatic hydrocarbons, and at the same time, building and improving fermenters, pioneering biphasic fermentations and integrating that with organic chemistry approaches to open up new synthetic pathways for complex chemicals. In this challenging environment, Bernard acted as coach, stimulator and visionary for his colleagues, collaborators and students, whom he granted a great learning and working environment and lots of room to explore new avenues. Quite unique in those days, he worked closely with companies and encouraged young entrepreneurs to start their own businesses [**Menno Kok (1980–1990)**].

One day, in the late 1980s, he was talking about the oxidation of hydrocarbons. At some point, he grabbed a napkin and drew the alkane oxidation pathway but with the arrows pointing in the wrong direction. Then he drew a tree around it and a fuel nozzle coming out of the tree. At this time, biofuels were not on anybody's mind yet – except for his. When I pitched Bernard my thoughts on cloning and characterizing a set of genes related to PHA synthesis as my graduate project, he dismissively said, ‘I have no idea why you would want to study that, but if you think there is something there, than go ahead’. My initial disappointment was immense. Yet suddenly my resolve was limitless. Years later, I realized that Bernard's brilliance as a mentor was to encourage a drive from within each of his students. In that instance, it took him under 5 min to get me completely ramped up for the next 4 years and beyond! [**Gjalt Huisman (1985–1991)**].

After finishing my major in Bernard's group under the supervision of Roland Lageveen, Bernard offered me a 6-month minor in North America in a group of my choice, and a PhD after my return. I didn't hesitate a second, and this was the start of an important period in my life, in which Bernard played a central role as mentor, and later as friend. As a mentor, he taught me to see no boundaries, unless people stop you with good reason. His relentless positive attitude was inspiring. I will never forget the first draft of my first scientific paper. ‘Great paper’, he said. Yet as I read through his mark-up I found hardly any of my own text left! He was able to find that balance of giving you freedom to experience and make mistakes, while coaching you so you didn't get lost. Bernard was a visionary thinker who paid attention to the people around him [**Hans Preusting (1985–1992)**].

As an illustration of Bernard's holistic view on biocatalysis, I was given the opportunity to do my major in biochemistry under supervision of Peter Terpstra in the group of Bernard to work on raising cofactor levels in bacteria. There were indications that for some reactions cofactor levels were limiting and this, in conjunction of overexpressing oxidative enzymes and maintaining cell viability in presence of reactants, needed to be addressed. After my major, Bernard brought me in contact with Ken Timmis, then professor in Geneva, where he gave me the opportunity to work on aromatic hydrocarbon conversions under the guidance of Shigeaki Harayama for a period of 9 months. When Bernard invited me to return to Groningen to do a PhD, we decided to add the aromatic biotransformations to the alkane bioconversions we had studied before, which opened a new field of research. I dearly remember Bernard as an inspirational supervisor with a sharp eye on the scientific background and a good nose for new opportunities. When he decided to move to the ETH in Zürich, to succeed Professor Armin Fiechter, the opportunity to bring lab results to actual processes was evident to Bernard. I was fortunate to join Bernard in this period, which was challenging and motivating at the same time. Bernard has always been able to build a strong, coherent group by providing appropriate guidance and providing room to explore your own path at the same time. His vision, personality and friendship remain a great inspiration to me [**Marcel Wubbolts (1985–1997)**].

I met Bernard first during a student excursion in Japan in 1984, where he invited me to join his Biochemistry department - MEMbranes group in Groningen. After a year in San Francisco, which he made possible, I started my thesis on the biochemistry and genetics of alkane degradation. In 1992, I moved with him to the ETH Institute of Biotechnology in Zürich and worked on alkane-degrading bacteria, biocatalysis and bioplastics until 2005 when Bernard retired. One of his outstanding characteristics was his great optimism, which sometimes made it quite challenging to talk him out of ideas. I learned a lot about finding alternative ways to approach problems, about using numbers as arguments, and about perseverance. Bernard always kept his optimism and his strong will to continue. The last time I met him – in January 2015 – he attended a lecture on the Hönggerberg in Zürich [**Jan van Beilen (1985–2005)**].

Bernard was an exceptional mentor and principal investigator. He believed that intricate scientific problems can best be tackled by recruiting excellent collaborators and endowing them with the maximum freedom to develop their project. In this way, he nurtured numerous scientific offspring into leading positions in industry and academia, where they continue to promote his integral view on biotechnology based on a critical evaluation of the scientific basis and a clear view of its quantitative potential of a field. He paved the way for a strong translation of biocatalysis from academia into industry. It is a tragedy that he has passed away, when so many of his ideas and concepts come to fruition outside of academia [**Sven Panke (1995–1999)**].

Bernard developed an integrated way and a complete scientific toolbox for chemical biotechnology. Thinking problems through to the end and focusing on the limiting issues, he integrated methodologies of microbiology, biochemistry, genetic engineering, biochemical engineering and economic efficiency for industrial applications in his biocatalysis cycle. Most importantly, this reflected the structure of his own laboratory at the Institute of Biotechnology (IBT) at ETH Zurich. Every lab member had the chance to travel through the biocatalysis cycle together with Bernard personally and scientifically, and found all the necessary infrastructure and support without any limitations. Many scientific questions were identified, developed and answered. One example covering everything is his contribution to alkane biocatalysis. Bernard set milestones for environmental microbiology, chemical biotechnology, bioplastic, toxicity, epoxides, redox biocatalysis, mass transfer, productivity, process integration and intensification, and finally, the transfer into industrial applications. Bernard, as head of the IBT, together with Jay Bailey and his laboratory, realized this vision under the mission ‘From DNA to Product’. The challenging, vibrating and lively environment resulted not only in numerous key scientific achievements of Bernard. It was also very influential and imprinting for me, so many people, and their future ways, not only in science but life in general [**Andreas Schmid (1996–2004)**].

Professors are usually evaluated in terms of number of publications and patents, and by the comparison of H-indices and impact factors. Being responsible for the recruitment of professors at ETH Zurich for more than 10 years, I know that the personality of a professor, the capability to do excellent teaching and to motivate students and co-workers, are just as important as scientific output. In this respect, Bernard did a fantastic job, and we are very grateful to him! He provided a collegial and stimulating research environment at the Institute, and he supported the development of individual careers by triggering our integration and recognition within the scientific community. Many of his former PhD students and post-docs are now academics or have leading positions in industry; others have started their own companies or are in prominent positions outside of science. A spark of Bernard's passion and exceptional spirit was transmitted and is still alive in many of us today [**Birgit Kessler (1994–2001)**].

‘Being a scientist’ was Bernard's great everyday joy. Conveying his love for science and of the severe scientific methodology (which involved many spreadsheets!) to his pupils was his natural state of being. And pupils of him we all were, whether undergraduate student, PhD student, or group leader. Still vivid in my memory is a day in May 1999 or 2000. In one of the ETH classic lecture halls, which was full of students, one of us group leaders was about to start a biotechnology lecture when Bernard came to the front and gave a seemingly unprepared but stunningly brilliant motivational speech on biotechnology in general. It provoked a spontaneous standing ovation. It's such a shame that this performance is not on tape. The career impact on the people with whom Bernard worked cannot be overestimated – and that includes me. His major role in my career was that of stimulating and developing my entrepreneurial interests, more specifically in making me realize that running your own business in a field in which you are good is the most natural and fulfilling thing a person can do in life. In so doing – in addition to all his practical input – he also stood at the basis of the present situation that the equipment and technology for miniaturized cultivation systems (that, under Bernard's guidance, we developed at the IBT at the end of the 1990s), now used in hundreds of biotechnology laboratories around the world [**Wouter Duetz (1997–2003)**].

I know Bernard as a kind, supportive and charismatic person with humour. When I joined his group as a PhD student, I had many concerns about taking on a research project without having any previous experience. Bernard took time from his busy schedule to learn about my anxiety and my academic interests. Several times when I interacted with him, he told me to stay strong in my beliefs and to continue working hard wherever I ended up. He reminded me that I was more than prepared to make something of myself. I never expected that, in time, these interactions would become one of my greatest sources of validation and confidence in my professional life [**Qun Ren (1993–1998)**].

Within the past two decades, Bernard grew from a mentor into a friend. My memory of him is that of a true gentleman, and of brilliant person who cared about mankind, this planet of ours and our future. I learned two very important things from him: (i) always calculate first! and (ii) nothing can stop a good idea. These two simple statements have guided me since our first meetings when I was a PhD student under his supervision [**Martin Held (1994–2002)**].

On the first day of our PhD theses, Roland Durner and myself were introduced by Bernard into the exciting world of medium chain-length PHA. Equipped only with a pen and a piece of paper, he explained to us the scientific background of our project – the biosynthesis of PHA in *P. oleovorans* under nutrient limitations in chemostats – which he had set up with Thomas Egli at EAWAG (Eidgenössische Anstalt für Wasserversorgung, Abwasserreinigung und Gewässerschutz). He did this so convincingly that Roland and I wanted to run immediately to the lab (and this happened later on several times)! With doubled enthusiasm, we soon identified the cultivation parameters – particularly the dual (carbon, nitrogen) limiting growth conditions – required to optimally produce PHA. I could exploit this with Bernard for the next 8 years, covering my postdoctoral time and as a junior group leader at EMPA (Eidgenössische Material Prüfungs Anstalt), where I had the pleasure to co-supervise with him my first PhD student. I am very grateful for the inspiring time I could share with him [**Manfred Zinn (1994–1998)**].

Bernard was a visionary, a philosopher and a technology leader. This combination reflected his expansive thinking and approach to the complex overlapping areas of biodegradable polymers and microbial biotechnology. His endeavours spanned from molecular microbiology to pilot scale PHA production. His positive influence on people was enormous, and from his laboratory emerged many of the current academic experts in PHA and microbial biotechnology. While Bernard was a highly successful academic scientist, to me, he was an entrepreneur. His philosophical disposition juxtaposed with his practical thinking. Bernard challenged himself and those who worked with him to innovate. Bernard broadened my horizons by showing me that innovation brought as many challenges and excitement as academic research. His philosophy of marrying fundamental research with practical scale-up and innovation resonated strongly with me. My time as a postdoctoral scientist under Bernard's leadership greatly influenced my decision to form a biotechnology company focusing on biodegradable polymers [**Kevin O'Connor (1997–1999)**].

One of Bernard's characteristics was his tendency to get involved in very competitive areas of research – in ‘trending topics’ both from a research and commercial point of view. A month before I joined Bernard's group, he invited me to attend a congress on bioplastics being held in Davos. I was shocked when I realized that the research line in which I was to invest my time as a post-doc in his laboratory lay in the sights of hundreds of researchers and businesses on every continent. During my PhD, I had carefully selected my objectives, cultivating an exclusive patch free of strong competitors that might publish results before me. Bernard reassured me, however, promising me that the enjoyment to be found in biotechnology was trying to bring to society what it required, that it provided well-being. He also assured me that companies would inevitably try to make money from our discoveries and not to worry about it. He thus helped me form part of an exciting world that requires being open to collaboration between the disciplines of molecular biology, biocatalysis, metabolic and chemical engineering, and metabolic modelling. Does this not remind you of the ‘Societal Challenges’ section of the current EU Research and Innovation programme Horizon 2020? I spent my last day in Zurich as a post-doc in Bernard's group in 1998, at his house, celebrating my leaving party with him and my colleagues. Over a glass of beer and appetizers, he talked about how, in the coming decades, we would need to look for wealth in our waste – the idea of valorizing our refuse that now forms the basis of bioeconomics. ‘You have a few years for making something out of this in your work, but then we'll discover how to capture energy easily from the sun – and everything will change’. I hope we'll be ready for it [**Auxi Prieto (1996–1998)**].

We will keep you in our hearts. Thank you very, very much, Bernard Witholt.

